# The Intra-Articular Combination of Fentanyl, Dexamethasone, Clonidine, Ropivacaine, and Dextrose to Treat Pain Due to Knee Osteoarthritis: A Case Report

**DOI:** 10.7759/cureus.64891

**Published:** 2024-07-19

**Authors:** Theofilos Tsoleridis, Alexandros Pittas

**Affiliations:** 1 Anesthesia and Pain Treatment Unit, General Hospital of Rhodes, Rhodes, GRC; 2 Department of Orthopaedics and Traumatology, General Hospital of Rhodes, Rhodes, GRC

**Keywords:** clinical case report, knee osteoarthritis/ koa, chronic pain management, multimodal pain management, intra-articular cocktail infiltration

## Abstract

We report a case involving the pain management of a patient with knee osteoarthritis (KOA), where conventional treatment failed to provide pain relief. Instead, a multimodal approach including an intra-articular (IA) injection of a combination of various agents was applied successfully. The pharmacological treatment resulted in minimal improvement. After experiencing failure with IA hyaluronic acid and platelet-rich plasma injections, an IA combination of fentanyl 50 mcg, dexamethasone 8 mg, clonidine 150 mcg, ropivacaine 7.5% 5 ml, dextrose 30% 5 ml, and normal saline 5 ml was applied. The treatment led to a two-year pain relief. The multimodal approach seems to offer satisfactory and encouraging results as the improvement in the quality of life led to favorable physical and psychological outcomes in the patient.

## Introduction

Knee osteoarthritis (KOA) is a common chronic, progressive, and degenerative disorder. It is characterized by inflammation and structural changes of the subchondral bone, leading to articular cartilage damage. KOA commonly affects women between the ages of 50 and 60 years and is one of the main causes of disability in patients over the age of 65 years. KOA can be categorized as primary (related to aging or lifestyle factors) or secondary (caused by several pathological conditions). While the etiology of KOA remains unclear, age, trauma, obesity, and joint overuse are considered to be significant risk factors [1]. Symptoms of KOA include pain, tenderness, a limited range of motion, joint effusion, and inflammation. The diagnosis is based on a triad of symptoms: stiffness, decreased function, and pain that worsens with activity but improves with rest. Also, crepitus, bony enlargement, and limited movement may be present. Radiography is the gold standard for diagnosis, but the correlation between severity and symptomatology is low [1].

The management of KOA primarily aims to decrease pain, alleviate disability, and preserve mobility and can be classified into pharmacological (analgesics and anti-inflammatory agents) and non-pharmacological (physiotherapy, biomechanical interventions, weight loss, etc.) approaches. If both regimens are ineffective, the patient may undergo total joint replacement (TJR) surgery [1]. Patients, who do not respond well to oral medication, and cannot undergo or prefer to postpone surgery, can benefit from various types of intra-articular (IA) knee injections, such as hyaluronic acid and platelet-rich plasma [1]. We discuss the case of a patient who did not attain pain relief through conventional treatment and received an IA combination of various medications after providing informed consent. There is scarce data in the literature on the simultaneous IA administration of the medications reported in this case.

This manuscript adheres to the applicable EQUATOR guideline, and written consent has been obtained from the patient for the publication of this case report.

## Case presentation

The patient was an 81-year-old female patient who visited the orthopedic clinic of the General Hospital of Rhodes, Greece in February 2020, complaining of excruciating and debilitating pain in her right knee. Her medical history highlighted hypertension, cardiac insufficiency, type 2 diabetes, and chronic obstructive pulmonary disease, which were being treated with furosemide (40 mg), carvedilol (12.5 mg), metformin (850 mg), and tiotropium (2.5 mcg), respectively. After clinical and radiology exams, she was diagnosed with KOA and prescribed paracetamol (two tablets of 500 mg) and diclofenac (one tablet of 50 mg). Given her complex medical history, the patient was advised to monitor her blood pressure, blood glucose, and oxygen saturation regularly, and to report anything that might concern her condition. One month later, she revisited the hospital with no change in symptoms. At this point, diclofenac was switched to tramadol/dexketoprofen (one tablet of 75 mg/25 mg), and she was discharged. After one month, the patient reported minimal improvement and was scheduled for IA hyaluronic acid injection.

In May 2020, the patient underwent five IA injections of hyaluronic acid (20 mg once per week) using a 22-gauge needle in a sterile manner. At the follow-up three months later, she reported an improvement that lasted for about one month, followed by a resurgence of her initial algological condition. As IA hyaluronic acid seemed to offer no results, the decision was made to switch to IA platelet-rich plasma. In September 2020, the patient underwent a single IA platelet-rich plasma injection; 30 milliliters of peripheral blood were taken in a sterile manner from the patient’s cephalic vein, which, after double plasma centrifugation, was enriched with 5 ml of concentrated platelets and injected into the patient in a sterile manner, using a 22-gauge needle. At the follow-up three months later, she reported a mild improvement that helped her regain the ability to perform some of her everyday activities. However, in February 2021, she revisited the hospital, complaining that her condition had worsened and that the improvements she had seen in the previous months had disappeared.

Apart from the pharmacological treatment failure, the patient was not eligible for either IA stem cell injection (due to age) or TJR, which was considered too risky given her condition. After careful evaluation and discussion with the patient, an IA combination of fentanyl (50 mcg), dexamethasone (8 mg), clonidine (150 mcg), ropivacaine 7.5% (5 ml), dextrose 30% (5 ml), and normal saline 0.9% (5 ml) was administered with the landmark-guided technique after obtaining her written consent. The patient was discharged and was scheduled for follow-up three months later. At the follow-up visit, the patient reported a remarkable improvement. Her pain had disappeared completely, and she was able to perform her everyday activities. At the second follow-up, six months after the injection, she no longer felt any pain and was able to perform her everyday activities without any difficulties. In February 2022, one year after the injection, she reported rare pain attacks that she could counter with paracetamol (one or two tablets of 500 mg).

In December 2022, the patient visited the hospital and reported that in the previous months, the pain attacks had reappeared. They were sporadic at first but became more frequent as time passed. Initially, she was able to manage her condition with paracetamol (two tablets of 500 mg) and diclofenac (one tablet of 50 mg) when she felt it necessary; however, in the last month, her condition had worsened, hindering her ability to perform everyday activities. While she described her condition as better compared to two years previously, she still could not bear the pain. She is now scheduled to undergo a second injection with the combination described above, as she indicated that she was satisfied with the result.

## Discussion

There are many studies in the literature on the intra-articular use of local anesthetics. Most of them have focused on postoperative pain following arthroscopic surgery, showing positive results and reporting no adverse effects. The most common IA local anesthetics are bupivacaine and ropivacaine [2]. Unfortunately, as reported in the study of Devi et al., local anesthetics have been generally found to exert chondrotoxicity and decrease chondrocyte viability in a dose-related manner [3]. Ropivacaine was chosen in this case because it is less toxic and seems to induce vasoconstriction locally, impairing plasma absorption and thus prolonging the analgesic effect [4].

As noted by Varkel et al., the existence of peripheral opioid receptors is well documented. Regarding IA opioid use, fentanyl (50 mcg) has exhibited a greater analgesic efficacy than morphine (3 mg). This is probably due to the high lipophilic properties of fentanyl. Moreover, fentanyl is not associated with the local release of histamine. Meanwhile, morphine seems to be linked to a dose-dependent release of histamine. Histamine (as the substance P) is associated with vasodilation and increased vascular permeability, which could lead to bradykinin release, resulting in a cascade of physiological and chemical changes that cause hyperalgesia [5]. Finally, fentanyl has generally been praised for its efficacy and low incidence of side effects when used to treat both postoperative pain and acute pain syndromes and plays a pivotal role as an analgesia supplement after knee arthroscopy when used intra-articular [6].

Clonidine, an alpha 2-adrenergic agonist, has been shown to be involved in peripheral analgesia by inhibiting nerve signal conduction through C and A-D fibers, thus inducing the release of encephaline-like substances, and by inhibiting the release of norepinephrine in nerve endings. If combined with local anesthetics, clonidine is indirectly involved in analgesia, and it has been shown to prolong their action. According to the meta-analysis by Sun et al., there have been many studies on IA clonidine efficacy in managing postoperative pain (again after arthroscopy), but the results concerning analgesia efficacy and duration have been contradictory. In the same study, clonidine exhibited a definite analgesic effect, which the authors described as mild, lasting, and antiemetic, but the mechanism was unclear. The authors also highlighted a possible hypotension risk, likely due to systemic clonidine uptake [7].

The IA use of dexamethasone has been well documented. It is a 9α-derivative synthetic glucocorticoid with a long biological half-life, high anti-inflammatory properties, and minimal mineralocorticoid activity. It has been shown that steroids generally block impulse transmission in the C fibers [8]. According to Grodzinsky et al., apart from its anti-inflammatory and analgesic actions, dexamethasone also protects the cartilage of the affected joint, normalizing the synthesis and release of glycosaminoglycans, decreasing proteoglycan and collagen loss, and preserving chondrocyte viability. Furthermore, dexamethasone prolongs the analgesic action of local anesthetics, and hence they are used in combination to treat postoperative analgesia and arthritis [9]. The IA use of dextrose has been reported to improve healing and pain control of the knee. According to Sit et al., many randomized controlled trials, systematic reviews, and meta-analyses have confirmed that IA dextrose leads to pain relief and functional improvements. While the exact mechanism of action remains obscure, basic science and initial clinical studies point to possible inflammatory cascade stimulation, a non-inflammatory proliferative effect, and chondrogenesis [10].

The approach regarding the treatment of this patient involved utilizing a multimodal approach to combat the algological condition, harnessing all the positive aspects of the intra-articular agents. As the patient was not eligible for intra-articular stem cells or TJR and was non-responsive to both pharmacological and invasive treatments, improvisation was required. The absence of pain for about two years led to significant improvement in her quality of life and helped the patient not only physically but also psychologically.

## Conclusions

The multimodal approach for managing knee osteoarthritis pain described above seems to offer satisfactory and encouraging results. The authors now plan to perform a randomized control trial by using the aforementioned agents to assess the results on a larger scale.
